# Exergy Efficiency Is a Key Performance Indicator to Rank Advanced Active Energy Technologies at the District Level

**DOI:** 10.3390/e28060693

**Published:** 2026-06-16

**Authors:** Daniel Favrat

**Affiliations:** Energy Center, Ecole Polytechnique Fédérale of Lausanne (EPFL), 1015 Lausanne, Switzerland; daniel.favrat@epfl.ch

**Keywords:** exergy, efficiency, district heating and cooling, heat pumps, cogeneration, SOFC, gas turbine, CO_2_ separation

## Abstract

Supplying electricity, heat and cold is an essential part of the development of more sustainable city districts. Previous studies have illustrated interest in using the exergy efficiency concept to decompose the problem and rank the technology combinations for heating or cooling according to their overall efficiency. This methodology is extended herein to include network losses that depend on the temperature level and grid losses that play a role when comparing electricity supplied by the grid rather than by local cogeneration units. This extended method is then applied by considering two emerging technologies. The first is very-low-temperature district heating and cooling (DHC), directly supplying air-conditioning needs via simple heat exchangers, as well as heating needs via local heat pumps. It is part of what are called fifth-generation DHCs or “anergy networks” and based on water or, better, on CO_2_ heat-transfer fluid. The main features of the five generations of networks, as well as the average aggregate user needs, are summarized in pinch technology composites, but replacing the temperature axis with a heat exergy axis to graphically highlight exergy losses. The average aggregate heating and cooling needs of users result from the application of a geographic information system to a district in a real city. The second emerging technology considers hybrid SOFC–GT cogeneration units, with or without CO_2_ separation, supplying electricity to all network users, including decentralized heat pumps, while optimizing the recovery of waste heat. The full synergy between heat providers and users is highlighted, allowing districts without cooling towers or chimneys except at one energy balancing plant. Integration of the advanced SOFC–GT cogeneration unit considered herein into the same anergy network allows an increase in exergy efficiency from 13.6% to 21.3%, as compared with electricity being supplied entirely from the grid and produced with a similar natural gas fuel.

## 1. Introduction

There is a strong consensus that the world needs to curb its greenhouse gas (GHG) emissions. As shown by the International Energy Agency (IEA) as early as in their 2009 outlook report [1], energy efficiency is key to achieving this goal. In that report, the relative contribution of increased efficiency was even more important than that of renewables, nuclear and Carbon Capture and Storage (CCS) considered separately. Of course, energy efficiency designates a very broad field, and major inefficiencies do exist today in many areas. One problem is the fact that energy efficiency is still too often based only on First Law considerations [2] (energy effectiveness), which do not consider the full potential of efficient use of resources. The main reason for this is the backlog of old technologies, like fuel boilers or second to third-generation nuclear reactors, contaminating the thinking of practitioners who are afraid of using more coherent indicators like exergy efficiency. As indicated in [3], domestic fuel boilers have exergy efficiencies on the order of 7%. Present nuclear reactors up to the third generation have exergy efficiency of less than 1%, considering the fuel potential lost in nuclear waste, which includes the minor actinides responsible for a large part of the waste’s radiotoxicity. A major part of this wasted exergy can be recovered in more advanced technologies, like fourth-generation molten salt reactors [4]. The latter operate at higher temperatures and close to atmospheric pressures, which is safer. Molten salt fission reactor technology was proposed as early as the 1960s at Oak Ridge. Chinese scientists recently reported substantial progress in their 2 MW molten thorium–uranium reactor prototype [5]. It is mentioned here because small modular reactors (MVRs) are increasingly considered at the district level, including to meet the growing needs of decentralized data centers. Some of the latter are even being established in the basements of large condominiums.

With a growing population living in cities, urban areas have high potential for exergy efficiency improvements. Global warming and heat island phenomena are reinforcing the need for cooling even in the central to northern areas of the northern hemisphere, where heating was the main concern until recently. The multiplication of data centers for AI (Artificial Intelligence) with high cooling needs is a major challenge, but also represents opportunities for waste heat recovery at the district level.

Independently of passive improvements to the building envelope, synergies between users through heating and cooling networks, as well as the exploitation of novel active technological approaches to co- or trigeneration, have a significant potential to improve the exergy efficiency and reduce pollution. A previous study [6] introduced a rigorous method to calculate the overall exergy efficiency of many technical alternatives to supply heating or cooling services. Heating technologies included common systems based on either direct electric heaters or local electrical heat pumps or district heating networks supplied by cogeneration or heat pump units, while also considering the origin of the electricity. To do that, the overall system was divided into a superstructure of four subsystems (power plant, district heating plant, building plant and room heat distributors). It was applied in a local law on energy, imposing this calculation on developers. To simplify their calculations, tables of exergy efficiencies for each subsystem were provided, along with technologies to choose from. This simple approach allowed the calculation of the overall exergy efficiency by multiplying the exergy efficiencies of the subsystems under consideration. The same approach was used for cooling technologies. It allowed for recommending the following rules based on thermodynamics: heat at the lowest temperature possible and cool at the highest temperature possible.

This study proposes an extension of the methodology to include an evaluation of the grid losses and of the network thermal losses, as well as its application to cases including two more advanced emerging technologies. One is a district heating and cooling (DHC) system using CO_2_ as the thermal transport fluid, and the second is a new hybrid fuel cell–gas turbine cogeneration unit including CO_2_ separation and an oxy-combustion post-burner. The quantitative application is done considering the average heating needs of an existing district, which are well described in the literature. The paper includes a dedicated section for each of the two emerging technologies, with a relevant literature review, followed by a section on the application of the methodology. The choice of the very-low-network emerging technology (Section 2) is made in view of its capability to supply some cooling directly while recovering most of the waste heat sources along the network. The choice of the emerging cogeneration technology (Section 3) is based on its particularly high efficiency and on its capability to efficiently separate and transport CO_2_ from fossil or green natural gas in a context of decarbonization.

## 2. District Heating (and Cooling) Networks

District heating and cooling (DHC) is expected to be of growing interest for limiting both local and global pollution while maintaining or further developing energy services. With the growing trend of electrification, trigeneration, i.e., combined heating, cooling and electricity generation, is also progressing.

**Figure 1:**
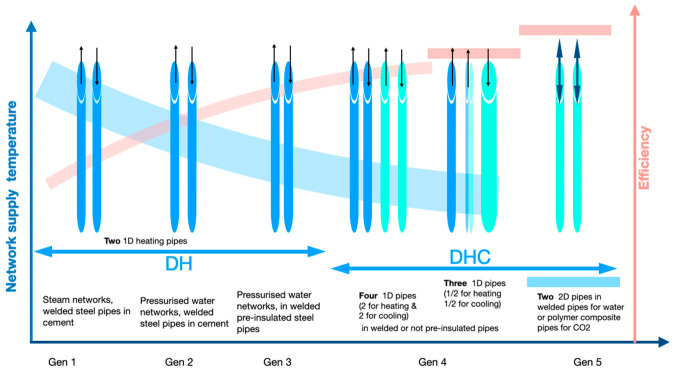
Generations of DH(C) networks (based on [3] and modified from [7]). The vertical arrows show the flow directions.

Industrialized countries and their cities have been, and still are, major contributors to greenhouse gas emissions. The first district heating (DH) systems began operation in the 1880s in the USA, using steam as the heat-transfer fluid. This technology was later developed by replacing the steam with pressurized water. The authors of [7] proposed a classification of DH(C) systems into four generations with a gradual transition from steam-based DH (Gen 1) to pressurized superheated water DH (Gen 2), to DH with unpressurized water in pre-insulated pipes (Gen 3), and, finally, to DHC networks integrating more energy functions, with a gradually decreasing temperature level (Gen 4) (Figure 1). Different papers, like in [8], focus on operational and reliability aspects of these networks supplying only heat, but those concerns are out of scope of the present study, which is limited to thermodynamic considerations of district heating and cooling networks.

A fifth generation has been introduced by several authors, but with different definitions, which has raised some controversy [9].

**Figure 2:**
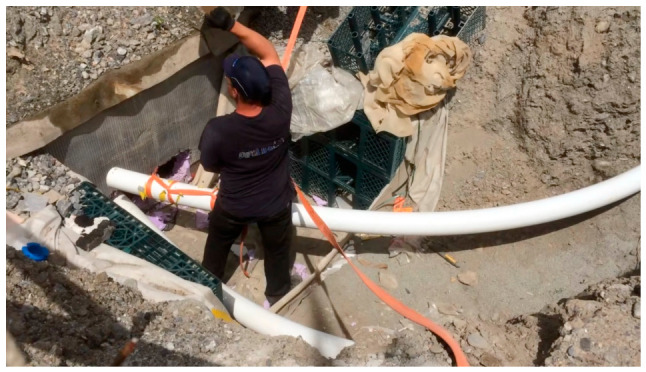
Composite pipe installation in a pilot CO_2_ DHC network in Sion (Switzerland) (photo by D. Favrat).

To be pragmatic, we propose the following definitions:

- Fourth generation: DHC with more than two one-directional pipes at relatively low operating temperatures (<70 °C). The most common are the following:
  ○ Fourth-generation networks include four pipes, two for heating and two for cooling.
  ○ Three-pipe networks can also be considered to be of the fourth generation, with one pipe switching temperature between winter and summer to better cope with needs [10]. A three-pipe system is, for example, used in the city of Nanterre in France [11], although only for heating with a 65 °C supply in one pipe, a 45 °C supply in a second pipe, and a single pipe for return flow to the heating plant. The latter is equipped with a heat pump and a separate piping system collecting low-temperature energy from sewage pipes to feed the evaporator. Another example is the EPFL cogeneration and heat pump energy plant [12] with two networks leaving the plant, one at 65 °C and one at 45 °C. Before being recently renewed, the plant was equipped with two NH_3_ heat pumps fed with lake water at the evaporator, plus a pipe delivering cold water from the lake for cooling services, with a simple discharge to the sewage system to avoid having a return pipe to the main plant. The same structure was mainly kept, with new NH_3_ heat pumps in a new building. However, this mode of operation with lake water supplied to the heat pumps’ evaporators, as well as directly for cooling, is being challenged by the recent quagga mussel proliferation that tends to invade lake water pipes.
- Fifth generation: Networks with two-pipe bidirectional networks operated close to ground-level temperatures, satisfying both heating via local heat pumps and cooling either directly or via local refrigeration units. This last generation of network, sometimes called *anergy networks* (anergy is the name given to the part of energy that cannot be converted into work, with the relation: energy = exergy + anergy. This term has been used to describe Gen 5 DHC networks, since these ones allow an easy use of waste or environmental heat). These networks can be subdivided into the following:
  ○ Water networks with small differences in temperature between supply and return, with the challenges of large pipes and significant pumping losses [13].
  ○ CO_2_ networks [14] using, at the customer side, the latent heat of this heat-transfer fluid without significant changes in the temperature level. One pipe is full of liquid CO_2,_ and the other is full of vapor CO_2_, both close to saturation. The pressure can then be used as a parameter to be optimized throughout the seasons. These DHC networks need to be pressurized at between 35 and 50 bars (saturation pressure) to be in the temperature range of 0 to 15 °C necessary to allow direct cooling services. Using latent heat, rather than the sensible heat of the transport fluid, results in a higher heat capacity per unit of volume flow and allows the use of smaller-diameter pipes for the same heat supply. As shown in Figure 2 from a demonstration DHC network [15], composite pipes from the offshore gas industry can be used in a compact assembly that does not require welding over relatively long distances. These pipes can be unrolled over several hundred meters. An extensive review of this class of network, including operational optimization on an existing district, is presented in [16].
- Marginal one-pipe water networks with one-way circulation and various supply sources and thermal storage, like that studied in Melbourne [17], could also be considered in the fifth generation. They rely on an energy balance between heat and cold users along the network, with thermal storage on the way and potential air towers (cooling when excess heat needs to be eliminated or supplying atmospheric heat to heat pumps for heating) to correct load imbalances. Fifth-generation DHC networks are also referred to as bidirectional low-temperature networks [13], low-temperature district heating and cooling networks [18] or balanced energy networks [19].

## 3. Hybrid SOFC–GT Cogeneration

In urban areas where the main needs are for electricity, heat and cold, cogeneration has been popular, mainly in connection with large DHC networks with relatively high supply temperatures typical of Gen 1 and Gen 2 DH. More recently, and on the lower power scale, Solid Oxide Fuel Cells (SOFCs) and their hybrid SOFC–gas turbine (GT) counterparts have emerged. These have significantly higher electrical efficiency and negligible health-affecting emissions. SOFCs, at their high temperature of operation, have the great advantage of letting only oxygen ions pass through the membrane from the cathode to the anode. They are therefore less sensitive to fuel quality. They also do not require expensive catalysts like those required for the lower-temperature proton-exchange fuel cells. They can be designed with either cylindrical or planar cells, although the latter have tended to dominate the trend in recent years. Pre-reformed natural gas, biogas or synthetic natural gas (SNG) is usually the fuel for SOFCs, since they can oxidize both hydrogen (H_2_) and carbon monoxide (CO). Considering that many cities are equipped with natural gas (NG) networks, these could be used to supply the cogeneration units instead of being deconditioned as part of the wave of decarbonization in cities. However, to compare with other decarbonization approaches, like those including the distribution of hydrogen, CO_2_ capture, and collection and transport to a point of use or disposal, are to be envisaged when natural gas is used. The latter is part of the features introduced in some of the concepts described below.

**Figure 3:**
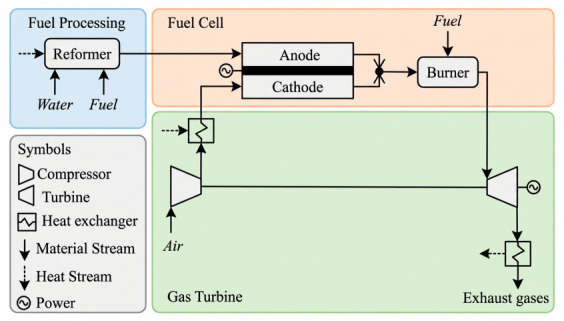
Hybrid SOFC–GT concepts with a pressurized SOFC (modified from [20,21]).

**Figure 4:**
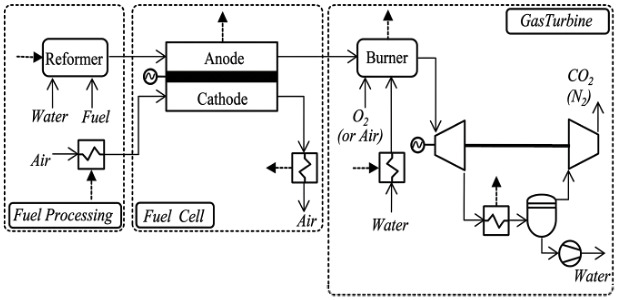
HC concept: hybrid SOFC–GT concept with an atmospheric SOFC and one sub-atmospheric gas turbine on the anodic exhaust (modified from [20,21]).

There are two main types of hybrid SOFC–GT. Figure 3 shows integration with a pressurized SOFC, which typically favors the use of an SOFC with tubular cells in view of the required pressure resistance. Occasionally, SOFCs using planar cells are envisaged, but they require a pressurized enclosure. In these concepts, the anodic and cathodic streams are too often mixed at the SOFCs’ exhaust. This impairs attempts to easily capture CO_2_ downstream by means of simple condensation, since nitrogen from the cathode is part of the exhaust gases. One significant point of interest of SOFCs is that they are inherently a separator of oxygen from the other components of air, like nitrogen. Only oxygen ions pass through the membrane, and the anodic exhaust flow then consists only of the oxidized fuel products. Therefore, it is important in these fuel cell designs to keep the anodic and cathodic streams separate at the exhaust. The anodic stream is made of steam, CO_2_ and remaining fuel, basically H_2_, in amounts that vary with the so-called fuel utilization factor. The latter is typically between 0.80 and 0.95. By capitalizing on this advantage, [20] introduced other concepts coupling atmospheric planar FC stacks with sub-atmospheric GTs (inverted Brayton cycle) and CO_2_ capture (Figure 4). The interesting part of the latter is that the inverted Brayton cycle consists of expanding the whole anodic flow in the turbine and, after steam condensation in a cooler, compressing only the remaining gas, mainly CO_2_. The condensed water is separately pumped, with a much lower energy requirement than would be required to recompress steam. Typically, the anodic gas exhaust temperature from the fuel cell is in the range of 780 to 900 °C. The remaining fuel in the anodic gas flow is typically burned in a burner that can be fed air or, preferably, pure oxygen to keep the CO_2_ and H_2_O concentrations in the gas as high as possible and favor efficient separation via condensation.

The advantage of the integration of SOFC or SOFC–GT cogeneration units with CO_2_ DHC is that the anodic exhaust flow can be cooled at the same low temperature all year round to favor maximum steam condensation. A high amount of condensation increases the electricity delivered by the GT and the “dryness” of the CO_2_ recovered.

Other concepts, including an additional sub-atmospheric gas turbine on the cathodic flow exit, were simulated and optimized in [21] and specifically examined for low-power units in [22].

**Figure 5:**
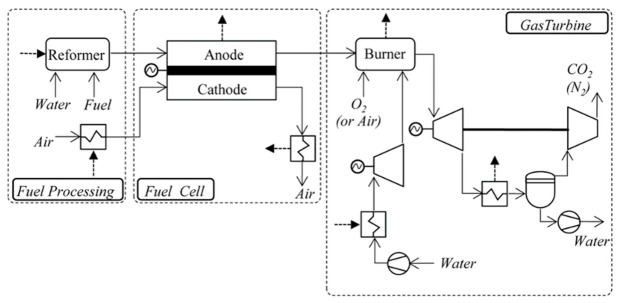
HCT concept: hybrid SOFC–GT plus Rankine cycle (modified from [20]).

Figure 5 shows another variation in the basic concept, designated HCT, in which elements of a Rankine cycle are added. These elements include a water feed pump pressurizing the water supply, a steam generator taking advantage of the heat recovered from the cathodic flow as it cools, and an independent steam turbine on the steam feed of the burner. The exhaust temperature of the burner can be controlled by the inflow of steam. Two burner exhaust temperatures of the flow to the sub-atmospheric gas turbine have been studied, as well as different pressure ratios for the GT. Table 1 compares the HC (Figure 4) and HCT (Figure 5) concepts for a GT pressure ratio of 3 and the highest turbine inlet temperature considered. Those results are based on a systematic process integration modeling approach and the use of an evolutionary optimization algorithm. The fuel cell model is based on a zero-dimensional model for planar SOFCs [23], calibrated using experimental data. The combustion in the burner is assumed to be stoichiometric and complete. The turbine and compressor are modeled with an isentropic efficiency of 85%. A pinch temperature difference of 10K is assumed. An exergy efficiency of 70% could be expected from the initial HC concept, and a further 8 percentage points could be achieved by the addition of the Rankine part (HCT concept).

**Figure 6:**
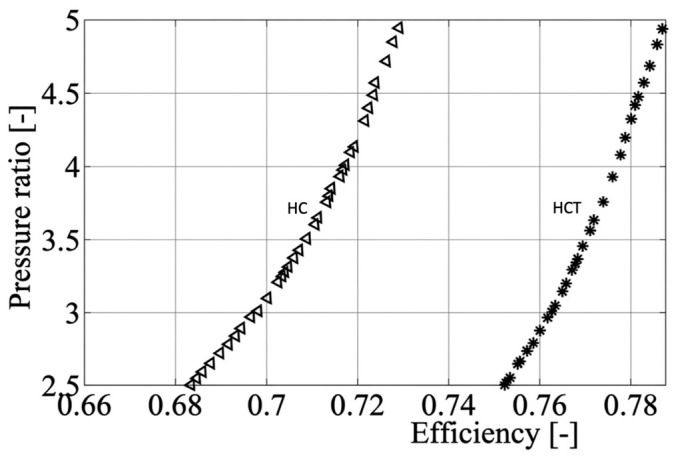
A comparison of the optimized designs for the HC and HCT concepts in terms of the pressure ratio (modified from [20]).

Figure 6 shows the results of the optimization of both concepts as a function of the pressure ratio. As can be seen, the exergy efficiency increases with the GT pressure ratio; however, for the methodological demonstration in this study, we chose a conservative value of 3 for further analysis of the integration with CO_2_ DHC. It is interesting to note in Table 1 the fact that the GT power fraction jumps from some 16% to more than 23% of the total power produced by the SOFC–GT assembly. Note that the exergy efficiencies of both concepts are already higher than those expected from a centralized natural gas power plant.

The exergy efficiency for the general cogeneration case with oxy-combustion in the burner, followed by CO_2_ separation and recompression to atmospheric pressure, can be written as follows:

(1a) $$\eta =\frac{{\dot{E}}_{GT}^{-}+{\dot{E}}_{SOFC}^{-}+{\dot{E}}_{Y\;CO_{2}}^{-}+{\dot{E}}_{Y\;water}^{-}-{\dot{E}}_{water\;pump}^{+}+{\dot{M}}_{CO_{2}}{\;e}_{d\;CO_{2}}}{{\dot{E}}_{y\;comb}^{+}\;+\;{\dot{M}}_{O_{2}}^{+}{\;e}_{d\;O_{2\;burner}}}$$

This equation uses the formalism adopted in [2], where ${\dot{E}}_{GT}^{-},{\dot{E}}_{SOFC}^{-},\;\mathrm{and}\;{\dot{E}}_{water\;pump}^{-}$ are electric power terms and ${\dot{E}}_{Y\;CO_{2}}^{-}\;\mathrm{and}\;{\dot{E}}_{Y\;water}^{-}$ are exergy transformation terms for the remaining water and CO_2_ flows leaving the integrated system. In fact, in [20,21,22], some of the numerator terms are already integrated in the reformer SOFC system, so the exergy efficiency, as calculated in Figure 6, can be simplified to become

(1b) $$\eta =\frac{{\dot{E}}_{GT}^{-}+{\dot{E}}_{SOFC}^{-}++{\dot{M}}_{CO_{2}}{\;e}_{d\;CO_{2}}}{{\dot{E}}_{y\;comb}^{+}+{\dot{M}}_{O_{2}}^{+}{\;e}_{d\;O_{2\;burner}}}$$

${\dot{E}}_{y\;comb}^{+}$ is the exergy transformation of the oxidation network and can be approximated by

(2) $${\dot{E}}_{y,comb}^{+}≅\;{\dot{M}}_{F}\;EXV\;$$

${\;e}_{d\;CO_{2}}$ and ${\;e}_{d\;O_{2\;burner}}$ are the exergy of diffusion of CO_2_ and O_2_. In the case of the HCT concept, the exergy terms of the Rankine-cycle part must be added to Equation (1).

For a generic fuel molecule $N_{F}\;C_{a}H_{b}O_{c}N_{d}$, oxidation with air corresponds to

(3) $$\frac{N_{CO_{2}}}{N_{Gc}}=\frac{a}{a+\frac{b}{2}+\frac{d}{2}+\left(a+\frac{b}{4}-\frac{c}{2}\right)(4.762\lambda -1)}={\tilde{c}}_{CO_{2}}^{Gc}$$

For natural gas, assimilated to methane CH_4_, and with an air factor λ=1,

(4) $$a=1,\;b=4,\;c=0,\;\mathrm{and}\;d=0;\;\mathrm{then},\;{\tilde{c}}_{CO_{2}}^{Gc}=\frac{1}{1+2+(1+1)(4.762-1)}=0.095$$

Hence, the theoretical exergy required to separate 10% CO_2_ from flue gas is

(5) $${\tilde{e}}_{dCO_{2}}=1\;\tilde{r}T^{0}ln\left(\frac{1}{{\tilde{c}}_{CO_{2}}^{Gc}}\right)=5831.9\frac{kJ}{{kmol}_{CO_{2}}}$$

This is informative since the real benefit (product) of such a cogeneration system, with CO_2_ separation, corresponds to the exergy of diffusion $e_{d\;CO_{2}}$:

(6)  ed CO2=e~d CO2m~CO2=20107.544=457 kJkgCO2

For comparison, Ref. [24] and Ref. [25] report energy requirements of 1297 kJ/kg_CO2_ and 1800 kJ/kg_CO2_ for actual processes, respectively. This tends to show that the present capture efficiencies given in the literature are not very high. Since 1 kmol of CH_4_ gives 1 kmol of CO_2_, the exergy of diffusion of CO_2_ corresponds to 1257 kJ/kg_CH4_, that is, 2.4% of the CH_4_ fuel’s exergy value. This might be considered low, but considering the present efficiencies of capture, these values are not negligible anymore.

Independent of the real capture efficiencies, it is also interesting to note that CO_2_ capture from the atmosphere requires close to 3.5 times more exergy than that from flue gas with 10% vol. CO_2_. In spite of this, CO_2_ capture directly from the atmosphere is often promoted.

When it comes to the supply of O_2_ for the burner, the lowest specific exergy needed corresponds to the exergy of diffusion ${\;e}_{d\;O_{2\;burner}}$.

For natural gas, assimilated to methane CH_4_, two kilomoles of O_2_ are required for complete combustion (2 times 3946.5), that is, 7893 kJ/kmol CH_4_. When compared with the exergy value of 830,174 kJ/kmol_CH4_, this represents only about 1%. This is, again, not a major part of the exergies considered, but it should be compared with the efficiency of O_2_ separation from the air in real processes. One way to analyze the supply of O_2_ to the burner, for practical consideration, would be to introduce another exergy efficiency for systems that include both oxy-combustion and CO_2_, as follows:

(7) $$\eta \prime =\frac{{\dot{E}}_{GT}^{-}\;+\;{\dot{E}}_{SOFC}^{-}\;+\;{\dot{E}}_{Y\;CO_{2}}^{-}\;+\;{\dot{E}}_{Y\;water}^{-}\;-\;{\dot{E}}_{water\;pump}^{-}\;+\;{\dot{M}}_{CO_{2}}{\;e}_{d\;CO_{2}}}{{\dot{E}}_{y\;comb}^{+}\;+\;{\dot{M}}_{O_{2}}^{+}{\;e}_{d\;O_{2\;burner}/\eta_{sep}}}$$

In this way, the efficiency of the cogeneration system is penalized to account for the processes required upstream to make pure O_2_ available. Note that a similar extension is not necessary in the numerical term related to the exergy services provided with CO_2_ capture, since the exergy required is already accounted for internally by the balance of the work terms. In practice, oxygen for the burner could be produced on site by the SOFC itself, since it has the capability to operate in reverse as a high-temperature electrolyzer when imported electricity prices are very low. In that operation mode, the O_2_ generated would need to be stored to be used later, when the SOFC operates again in cogeneration mode. This has not yet been accounted for in the integrated cases discussed in Section 4.

An extensive review of most of the processes described above regarding hybrid fuel cell–GT systems is presented in [26]. Its authors also provide a comparison of the simulated values for different SOFC–GT concepts and the few experimental data available. Contrary to what has been observed for many other power technologies, they do not identify a clear increase in the electrical effectiveness of hybrid SOFC–GT systems within the power range of interest in this study, i.e., a few kW to a few MW. Surprisingly, they observe that experimental data on electrical effectiveness (First Law) are so far around 10 to 15% lower than the simulated values. However, a demonstration project including the HC concept [19] and CO_2_ methanation for seasonal storage has recently been launched in Sion (Switzerland) [27]. This happens to be on the same site as the CO_2_ network cited earlier [15], with the objective, among others, of demonstrating the efficiencies quoted in the present paper.

## 4. Efficiency of Heating and Cooling Supply to a District

The interest in coupling a fifth-generation DHC CO_2_ network with an advanced SOFC–GT is linked to the following synergies:

(a) Fifth-generation DHC distributes heat at a temperature level that requires the use of essentially electrically driven heat pumps in each building or group of buildings. The DHC network provides a low-temperature sink for waste energy recovery all year round, as well as for cooling flue gas from the SOFC–GT while efficiently capturing CO_2_ from the H_2_O-CO_2_ stream of the anodic flow. The latter improves the inverted Brayton cycle’s contribution.
(b) Decentralized and pollution-free electricity meeting the needs of the district, including for the individual heat pumps, can be generated through CO_2_ capture. The captured CO_2_ can be processed as follows:
  - Either compressed at the level of the DHC pressure (around 50 bars) and transported by the network to a central collection plant;
  - Or, better, transported at a lower pressure to the central collection plant via either an available annular inter-pipe space or a third pipe. An annular inter-pipe space would be available if an external safety envelope is required by city regulations.

The collected CO_2_ can then be purified and stored in liquid form for summer use to generate synthetic natural gas by combining it with H_2_ from a district electrolyzer using excess solar electricity. Another interesting use of CO_2_ would be to combine it with a thermo-electric energy storage system (sometimes called a Carnot Battery) based on trans-critical CO_2_ cycles as proposed in [28].

**Figure 7:**
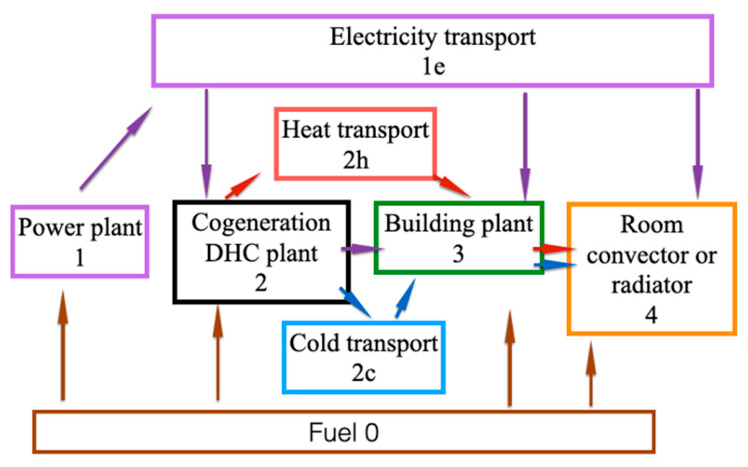
System decomposition for exergy evaluation [3].

Obviously, heat, cold and electricity can be provided using various technologies, but technology combinations perform best. The methodology applied in [6] introduced four main subsystems, numbered 1 to 4 in Figure 7. It aimed at rigorously answering basic questions, like why it is recommended to use low-temperature radiators or floor heating rather than traditional higher-temperature radiators or electric radiators to heat a room. It also questioned why it is better to distribute a cooling fluid as close as possible to the desired room temperature. Subsystems 1e, 2h, and 2c are added to include the exergy efficiency of transport and, therefore, account for the exergy losses. The present study adds technologies for CO_2_ separation and oxy-combustion.

As was shown in [6], the ideal is to formulate the modeling in such a way that the result for the complete system is the multiplication of the exergy efficiency of each subsystem. This facilitates the exergy ranking of technological options, integrating several technologies from the potential power plant, if considered, to the final user. However, as will be shown below, the integration of the two new emerging technologies will require a more sophisticated approach with energy and exergy balances of each subsystem.

**Figure 8:**
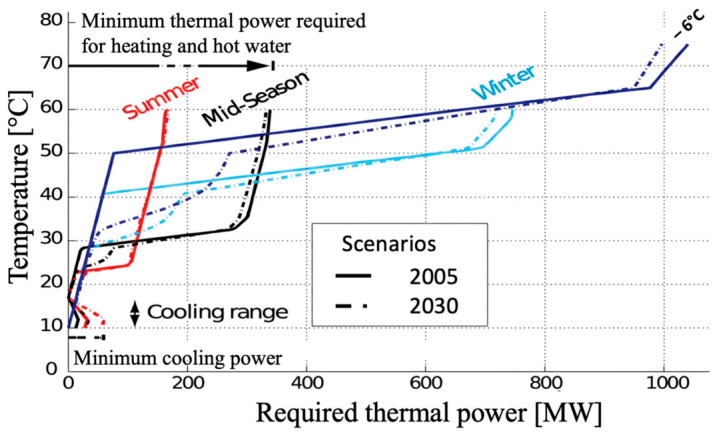
Energy composites for a city [29].

The efficiencies for different generations of DH or DHC can be compared using energy and exergy (Carnot) composites. The authors of [29,30] proposed structuring heating and cooling data using such composites in Figure 8. Too often, the energy needs of communities are only reported in terms of heat rates; energy composites allow us to account for the temperatures required by the heat distribution systems inside each building. This parameter is vital to judging the opportunities for heat pumps and particularly important in districts that have a mix of recent and old buildings.

**Figure 9:**
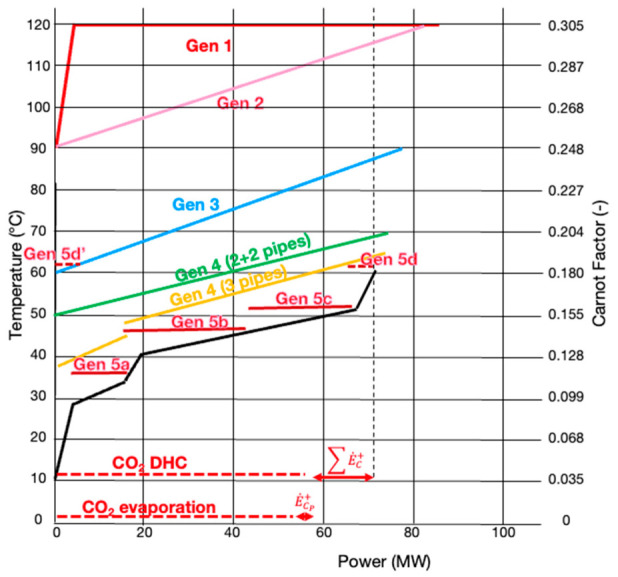
Energy composites (left-hand scale) or Carnot composites (right-hand scale) for the average winter case in [29], including the streams relative to the various generations of DH or DHC [3]. The average atmospheric temperature is assumed to be 0 °C.

Figure 9 shows the energy composites associated with the different generations of DH or DHC based on the average winter needs considered in Figure 8, but for 1/10 of the city heat rates quoted in [29]. If a Carnot factor is substituted for the temperature in the vertical axis, the area underneath each composite represents the heat exergy to be considered, and we may talk about exergy (or Carnot) composites. The areas between each DH composite (hot composite) and the exergy composite of the heating needs (cold composite, represented here as a black solid line) provide a graphical visualization of the exergy losses. The line with steps (a, b, c, d, d′) is an approximate representation of the hot composite resulting from a fifth-generation DH, since local heat pumps can adjust the condensing temperature to the actual needs of each building with small pinch differences. Demand 5a is from modern floor-heating houses, 5b is from retrofitted buildings, 5c is from older buildings, and 5d and d’ are for the lower and upper parts of hot water heating.

In this particular case, CO_2_ liquid and vapor are distributed at 12 °C. This temperature is maintained by an open heat pump at the balancing central plant (2). At that plant, in winter, the incoming liquid CO_2_ is first expanded, then evaporated and compressed to be supplied to the vapor pipe of the DHC. This setup allows for adaptation of the evaporation temperature to the actual source temperature. In this case, lake water is considered a heat source at 8 °C and cooled to 3 °C through CO_2_ evaporation at 1 °C. Power ${\dot{E}}_{CP}^{+}$ is needed for the CO_2_ balancing plant compressor. The sum of powers supplied to the building heat pumps along the network is designated $\sum {\dot{E}}_{C}^{+}$, and CO_2_ DHC indicates the sum of heat rates provided by the DHC to the building heat pumps.

**Figure 10:**
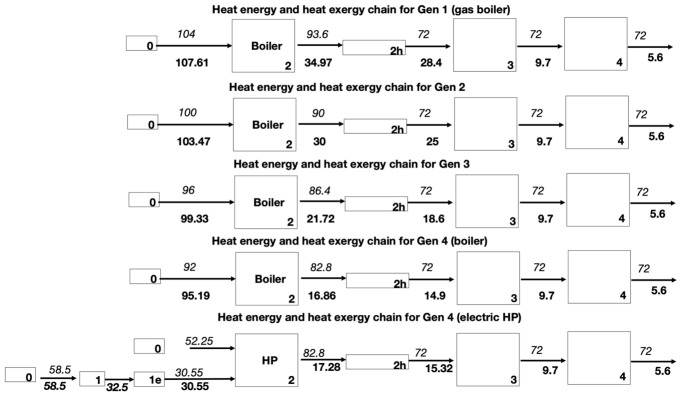
Block diagram examples of Gen 1 to 4 DH supplying average winter needs corresponding to the composite in Figure 9 (regular font for energy and bold font for exergy) [2,3]. The lower heating value is used for the energy values.

**Figure 11:**
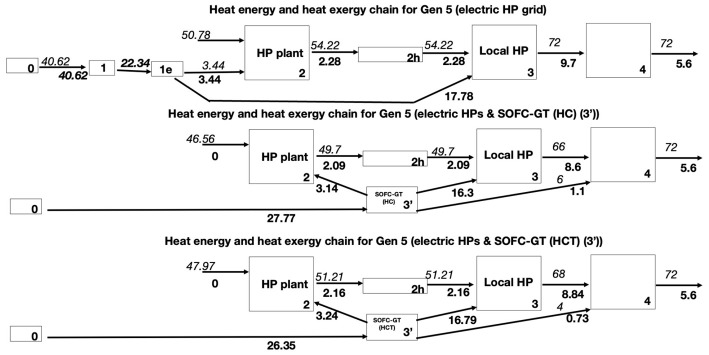
Block diagram for Gen 5 DH, including SOFC–GT cogeneration units (HC and HCT) supplying the same average winter needs (regular font for energy and bold font for exergy).

Figure 10 and Figure 11 show the different energy and exergy values for meeting the average winter needs of the part of the city considered in [29,30]. The first four are based on a gas boiler heat source. They differ according to their different levels of temperature distribution. The assumed energy losses vary with the DH supply temperature, as can be seen in Table 2.

No heat loss is considered for Gen 5 since the temperature distribution is close to the ground temperature. The other networks’ energy losses were chosen to be proportional to the temperature level. Note that these should be adjusted when considering a real network, because other factors, like the length of the network, should also be considered. The exergy efficiency of the local HP (COPth/COP) is an average value based on the literature. These are usually single-stage; therefore, their efficiency is lower than that of district-centralized heat pumps, which are often based on multistage cycles. The considered grid losses are in the lower range of existing grid losses, which can vary from country to country.

Figure 11 introduces cogeneration SOFC–GT units along the DH system with electric efficiency of 71% and 21.6% heat recovery at the same location. Table 3 provides the exergy efficiency of each technology used. For generations 1 to 4, the global exergy efficiency is the product of all exergy efficiencies along the various processes. For generation 5, this is no longer possible since different parallel processes intervene.

The net result shows the benefit of decreasing the FH supply temperature, with exergy efficiencies that remain low when the supply is based on a fossil fuel boiler.

Gen 5 solutions dominate among the alternatives for heating, even without considering the heat recovery from other sources along the network, like waste heat from data centers, office building air conditioning, and refrigeration and air conditioning in shops. Furthermore, direct free cooling would further improve these figures. Internal cogeneration with advanced SOFC–GT dominates among the considered alternatives. Being integrated into a CO_2_ network allows the system not only to efficiently separate CO_2_ but also to collect it and transfer it to a balancing plant that can act as a collection and storage point.

## 5. Conclusions

This study illustrated an extension of a previously published methodology to include grid losses and network heat losses. It showed the benefits of coupling an advanced fifth-generation DHC, based on CO_2_ as the heat-transfer fluid, with two different concepts of hybrid SOFC–GT cogeneration: CO_2_ capture and oxy-combustion. The methodology used to properly define exergy efficiency in these systems was discussed, and the benefit of lowering the district heating temperature was highlighted, including a graphical representation. Finally, a methodology was provided to coherently rank all alternative technologies in terms of their exergy efficiency. Different generations of district heating networks are compared. This study highlights the particularly low exergy efficiencies of the present boiler-fed district heating systems and the significant improvement in terms of efficiency that could be expected from emerging technologies. Exergy efficiency formulations for cogeneration systems with CO_2_ separation and oxy-combustion were proposed, and the relative energy requirements for CO_2_ separation were discussed. The approach provides a framework for further detailed studies to analyze real projects, which will need to include, among others, a finer decomposition into time slices and the inventory of waste heat sources along the network. Further work is required to elaborate on the new extended method to a broader range of heating and cooling technologies.

## 6. Patents

There are a few patents dealing separately with CO_2_ DHC or hybrid SOFC–GT concepts, but the focus of this study was to illustrate the proposed general methodology for comparing and ranking various DHC technologies on a thermodynamic basis.

## Figures and Tables

**Table 1:** Comparison between optimized HC and HCT concepts (modified from [20]).

Hybrid SOFC–GT Type	HC	HCT
Pressure ratio	3	3
Steam-to-carbon ratio	1.43	1.45
Steam reforming T [K]	1064	1053
Fuel cell T [K]	1072	1071
Fuel cell excess air	6.7	5
Fuel utilization	0.8	0.8
Steam T [K]	955	971
Inlet turbine T [K]	1573	1573
Compressor inlet T [K]	299	300
Exergy efficiency	0.698	0.78
GT power fraction [%]	15.7	23.5

**Table 2:** Main assumptions.

	Gen 1	Gen 2	Gen 3	Gen 4	Gen 4 HP	Gen 5
Energy losses [%]	30	25	20	15	15	0
Boiler effectiveness	0.9	0.9	0.9	0.9		
District HP (COPth/COP)					0.55	0.55
Local HP (COPth/COP)						0.45
Losses of main electric grid [%]	5	5	5	5	5	5

**Table 3:** Exergy efficiency results for different heating technologies meeting the average winter demand corresponding to Figure 4.

	$\eta_{1}$	$\eta_{1e}$	$\eta_{2}$	$\eta_{2h}$	$\eta_{3\prime }$	$\eta_{3}$	$\eta_{4}$	η
Gen 1			0.325	0.813		0.341	0.577	0.052
Gent 2			0.290	0.833		0.388	0.578	0.054
Gen 3			0.219	0.857		0.521	0.578	0.056
Gen 4			0.177	0.885		0.650	0.578	0.059
Gen 4 HP	0.556	0.940	0.566	0.887		0.632	0.578	0.096
Gen 5	0.549	0.952	0.663	1.000		0.480	0.577	0.138
Gen 5 HC			0.67	1.00	0.74	0.47	0.577	0.202
Gen 5 HCT			0.67	1.00	0.80	0.47	0.585	0.213

## Data Availability

The main data used can be found in the referred PhD theses that are freely available on demand to author.

## Abbreviations and Nomenclature

The following abbreviations are used in this manuscript:

DH	District heating	
DHC	District heating and cooling	
SOFC–GT	Hybrid Solid Oxide Fuel Cell–gas turbine	
GIS	Geographic Information System	
GHG	Greenhouse gas	
IEA	Internal Energy Agency	
CCS	Carbon Capture and Storage	
SNG	Synthetic natural gas	
HC	Concept of hybrid SOFC–GT fuel cell with one GT on anodic flow	
HCT	HC concept with additional steam turbine	
${\dot{E}}_{GT}^{-}$	[kW]	Net power (exergy rate) from gas turbine (GT)
${\dot{E}}_{SOFC}^{-}$	[kW]	Net power (exergy rate) from Solid Oxide Fuel Cell (SOFC)
${\dot{E}}_{Y\;CO_{2}}^{-}\;$	[kW]	Exergy transformation rate of CO_2_
${\dot{E}}_{Y\;water}^{-}$	[kW]	Exergy transformation rate of H_2_O
${\dot{E}}_{water\;pump}^{+}$	[kW]	Power (exergy rate) supplied to pump condensed water
${\dot{E}}_{y\;comb}^{+}$	[kW]	Exergy transformation of combustion
EXV	[kJ/kg]	Exergy value of fuel
${\;e}_{d\;CO_{2}}$	[kJ/kg]	Specific exergy of diffusion of CO_2_
${\;e}_{d\;O_{2\;burner}}$	[kJ/kg]	Specific exergy of diffusion of O_2_
${\dot{M}}_{F}$	[kg/s]	Mass flow of fuel
${\dot{M}}_{CO_{2}}$	[kg/s]	Mass flow of CO_2_
$N_{CO_{2}}$	[kmol]	Number of kilomoles of CO_2_
$N_{Gc}$	[kmol]	Number of kilomoles of exhaust gas
${\tilde{c}}_{CO_{2}}^{Gc}$	[-]	Molar fraction of CO_2_ in exhaust gas
λ	[-]	Air factor
$\tilde{r}$	[J/(K kmol]	Molar universal gas constant
η	[-]	Exergy efficiency
